# Solasodine, a Natural Steroidal Alkaloid, Attenuates RANKL‐Induced Osteoclastogenesis and Bone Resorption: A Study Based on Network Pharmacology and Experimental Validation

**DOI:** 10.1111/jcmm.71253

**Published:** 2026-06-20

**Authors:** Yiwei Jiang, Zhiyu Jin, Xiaoyi Ji, Maihuan Wang, Zhen Cao

**Affiliations:** ^1^ School of Medicine South China University of Technology Guangzhou China; ^2^ Department of General Surgery The Sixth Medical Center of Chinese PLA General Hospital Beijing China; ^3^ Department of General Surgery The First Medical Center of Chinese PLA General Hospital Beijing China

**Keywords:** NFATc1, osteoclastogenesis, osteoporosis, RANKL, solasodine

## Abstract

Osteoporosis is characterized by excessive bone resorption driven by aberrant osteoclast activation. Solasodine (SOL), a natural steroidal alkaloid, has undefined roles in bone metabolism. This study investigated SOL's effects on RANKL‐induced osteoclastogenesis and its underlying mechanisms. Pharmacological targets predicted via network pharmacology and validated by molecular docking identified 81 overlapping targets, which were primarily enriched in MAPK, NF‐κB, and JAK–STAT pathways, confirming robust affinity between SOL and core targets including NFκB1, JAK1/2, and STAT3. In vitro, bone marrow‐derived macrophages (BMMs) were stimulated with M‐CSF and RANKL. Evaluation via TRAcP staining, F‐actin immunofluorescence, and hydroxyapatite assays showed that SOL dose‐dependently inhibited RANKL‐induced osteoclast formation, fusion, and resorptive activity without cytotoxicity. Mechanistic investigations through RT‐qPCR, Western blotting, luciferase assays, ROS detection, and live‐cell calcium monitoring revealed that SOL suppressed key markers, including NFATc1, c‐Fos, CTSK, Atp6v0d2, and Integrin β3. Specifically, SOL attenuated MAPK (p38, JNK, ERK) and STAT3 phosphorylation, inhibited NF‐κB activity, and prevented IκB‐α degradation. Furthermore, SOL curtailed RANKL‐induced ROS generation, intracellular calcium oscillations, and subsequent CaMKIV activation. Ultimately, SOL inhibits RANKL‐induced osteoclastogenesis and bone resorption by suppressing the NFATc1/c‐Fos axis through coordinated modulation of the MAPK, NF‐κB, and JAK–STAT pathways, alongside mitigation of ROS production and calcium signalling, representing a promising natural candidate for treating osteolytic bone diseases.

## 1 Introduction

Osteoporosis (OP) is a systemic metabolic bone disease characterized by low bone mass, microarchitectural deterioration of bone tissue, and increased bone fragility [1]. Under physiological conditions, bone remodelling is maintained through a delicate equilibrium between osteoblast‐mediated bone formation and osteoclast‐mediated bone resorption; however, an imbalance in this homeostasis—particularly the aberrant activation of osteoclasts leading to excessive resorption—triggers progressive bone loss [2]. Currently, bisphosphonates serve as the first‐line clinical intervention for OP, yet their long‐term administration is associated with challenges such as osteonecrosis of the jaw, atypical fractures, and poor patient compliance [3]. Alternatively, while traditional steroidal hormonal therapies demonstrate established efficacy in maintaining bone density, their widespread clinical application is hindered by systemic endocrine disruption and potential risks of tumorigenesis [4]. Consequently, identifying novel bioactive molecules capable of precisely modulating bone resorption pathways with high safety has become a pivotal task in bone metabolism research. In this context, plant‐derived steroidal compounds have garnered significant attention due to their unique pharmacological activities. Unlike synthetic hormones, natural steroidal alkaloids often intervene in cellular signal transduction via non‐genomic pathways, thereby exerting regulatory effects while circumventing the typical side effects associated with conventional hormonal drugs [5]. Given our group's extensive experience in investigating the diverse pharmacological activities of Traditional Chinese Medicine (TCM) [6, 7, 8, 9], this study focuses on the therapeutic potential of natural compounds in bone metabolism.

As the primary executors of bone resorption, the formation and activation of osteoclasts are predominantly driven by the Receptor Activator of Nuclear Factor‐κB Ligand (RANKL) signalling axis [10]. The binding of RANKL to its receptor, RANK, triggers a cascade of complex intracellular signalling networks, including the classical mitogen‐activated protein kinase (MAPK) and nuclear factor‐kappa B (NF‐κB) pathways, as well as the JAK–STAT pathway, which has recently been identified as a critical regulator of osteoclast maturation [11, 12]. Furthermore, the generation of intracellular reactive oxygen species (ROS) and calcium oscillations (Ca^2+^ oscillations) act as essential synergistic signals during osteoclastogenesis [13, 14], promoting maturation by amplifying the cascade of the master transcription factor, nuclear factor of activated T‐cells cytoplasmic 1 (NFATc1) [14, 15]. This intricate multi‐pathway network suggests that effective therapeutic interventions must possess multi‐target regulatory capabilities.

Solasodine (SOL), a representative of these compounds, is a natural steroidal alkaloid widely found in the Solanaceae family. In the pharmaceutical industry, SOL serves as an essential precursor for the semi‐synthesis of various steroidal hormonal drugs [16]; this natural steroidal scaffold imparts unique physicochemical properties to the molecule. Recent pharmacological investigations have underscored the multi‐dimensional therapeutic potential of SOL across diverse pathological models. Specifically, SOL has been shown to attenuate Th2‐mediated airway inflammation and remodelling by modulating the Runx3/NLRP3 signalling axis [17]. In the field of oncology, SOL exerts potent anti‐metastatic effects by activating AMPK and subsequently inhibiting the STAT3 and NF‐κB pathways [18]. Furthermore, SOL exhibits significant neuroprotective activity by bolstering endogenous antioxidant defences and reducing oxidative‐stress‐induced damage [19]. Despite its steroidal backbone and multi‐targeted signalling potential, the specific impact of SOL on bone metabolism—particularly its effect on RANKL‐induced osteoclast differentiation and bone resorptive function—remains to be systematically investigated. Exploring whether SOL can intervene in the RANKL‐mediated signalling network to inhibit osteoclastogenesis holds significant scientific merit and clinical translational value.

## 2 Materials and Methods

### 2.1 Reagents and Materials

Fetal bovine serum (FBS), α‐Modified Eagle Medium (α‐MEM), Rhodamine‐Phalloidin, TRIzol reagent, and the Calcium Oscillation Assay Kit were procured from Thermo Fisher Scientific (Waltham, MA, USA). SOL (purity > 98%) was purchased from MedChemExpress (MCE; Monmouth Junction, NJ, USA). A stock solution of SOL was prepared by dissolving the powder in dimethyl sulfoxide (DMSO) and subsequently diluted in phosphate‐buffered saline (PBS) to a final concentration of 1 mM with a final DMSO concentration below 0.1% (v/v). Primary antibodies against NFATc1, c‐Fos, CTSK, Atp6v0d2, Integrin β3, β‐actin, JNK, p‐JNK, ERK, p‐ERK, P38, p‐P38, STAT3, p‐STAT3, CAMKIV, p‐CAMKIV, IκB‐α, NRF2, KEAP1, HO‐1, and NQO1 were sourced from Abcam (Cambridge, UK). The MTS assay and luciferase reporter systems were acquired from Promega (Madison, WI, USA). Recombinant murine RANKL and M‐CSF were supplied by R&D Systems (Minneapolis, MN, USA). The TRAcP staining kit and 4′, 6‐diamidino‐2‐phenylindole (DAPI) were obtained from Sigma‐Aldrich (St. Louis, MO, USA).

### 2.2 Primary Cell Isolation and Culture

Bone marrow‐derived macrophages (BMMs) were harvested from the long bones of 6‐week‐old C57BL/6J mice. Cells were maintained in complete α‐MEM containing 10% FBS, 2 mM L‐glutamine, 100 U/mL penicillin, and 100 μg/mL streptomycin, supplemented with 50 ng/mL M‐CSF. RAW264.7 cells, obtained from the American Type Culture Collection (ATCC; Manassas, VA, USA), were cultured under identical conditions. All cultures were kept in a humidified incubator at 37°C with 5% CO_2_.

### 2.3 Network Pharmacology and Molecular Docking

#### 2.3.1 Target Identification

Potential pharmacological targets of SOL were predicted using the SEA, SUPERPRED, HERB, and SwissTargetPrediction databases. Osteoporosis‐associated genes were identified through OMIM, DisGeNET, and GeneCards. The intersection of compound targets and disease genes was determined via Venny 2.1.0.

#### 2.3.2 PPI Network Construction

A Protein–Protein Interaction (PPI) network was established using the STRING database (interaction score > 0.4) and modelled in Cytoscape (v3.9.1). Topological attributes, including Degree, Betweenness, and Closeness, were evaluated using the CytoNCA plugin. Nodes exceeding the median value for all three parameters were screened as core hubs.

#### 2.3.3 Enrichment Analysis

Gene Ontology (GO) and Kyoto Encyclopedia of Genes and Genomes (KEGG) analyses were conducted via Metascape (*p* < 0.05). The resulting data were plotted in R (v4.2.0) utilizing clusterProfiler and ggplot2 for visualization.

#### 2.3.4 Molecular Docking

Three‐dimensional structures of JAK1, JAK2, STAT3, MAP3K7, MAP3K14, NFKB1, and TLR4 were retrieved from the RCSB PDB. Receptor and ligand preparation were performed using AutoDockTools (v1.5.6). Docking simulations were executed with AutoDock Vina (v1.1.2). Stable binding conformations were analysed through the PLIP server and rendered using PyMOL.

### 2.4 In Vitro Osteoclastogenesis Assay

BMMs were distributed into 96‐well plates (6 × 10^3^ cells/well) and stimulated with M‐CSF (50 ng/mL), RANKL (100 ng/mL), and various concentrations of SOL (0, 1, 2.5, 5, 10 μM). Following a 5 day induction period, cells were fixed with 4% paraformaldehyde and subjected to TRAcP staining. Mature osteoclasts, defined as TRAcP‐positive cells possessing three or more nuclei, were quantified by light microscopy.

### 2.5 Cytotoxicity Assessment

The effect of SOL on cell viability was determined via the MTS assay. BMMs were seeded at a density of 6 × 10^3^ cells/well and exposed to SOL (0–15 μM) for 48 h. Subsequently, 20 μL of MTS solution was added per well, followed by a 2 h incubation. Absorbance was recorded at 490 nm using a Multiskan Spectrum microplate reader (Thermo Fisher Scientific).

### 2.6 Confocal Immunofluorescence

BMMs were induced toward the osteoclast lineage as described. Post‐differentiation, cells were permeabilized with 0.1% Triton X‐100 and blocked using 3% BSA. F‐actin structures were stained with Rhodamine‐Phalloidin (45 min), while nuclei were counterstained with DAPI. Fluorescence images were acquired using a confocal laser scanning microscope.

### 2.7 Hydroxyapatite Resorption Assay

BMMs were pre‐differentiated on 6‐well plates. Mature osteoclasts were then transferred onto Corning Osteoassay plates (Corning, NY, USA) and treated with SOL (0, 5, 10 μM) for an additional 48 h. The resorption area was visualized via TRAcP staining and quantified using ImageJ software (NIH, Bethesda, MD, USA).

### 2.8 Quantitative Real‐Time PCR (RT‐qPCR)

Total RNA was isolated from treated BMMs using TRIzol reagent. One microgram of RNA was reverse‐transcribed using M‐MLV reverse transcriptase. PCR amplification was carried out using SYBR Green PCR Master Mix (Thermo Fisher Scientific). Transcript levels were normalized to ACTB and calculated using the 2^−ΔΔCt^ method. Primer sequences are presented in Table 1.

**TABLE 1:** Primer sequences for RT‐qPCR.

Genes	Forward (5′‐3′)	Reverse (5′‐3′)	*T*_m_ (°C)
*Hmbs*	AAGGGCTTTTCTGAGGCACC	AGTTGCCCATCTTTCATCACTG	60
*Nfatc1*	GACCCGGAGTTCGACTTCG	TGACACTAGGGGACACATAACTG	60
*Fos*	GCGAGCAACTGAGAAGAC	TTGAAACCCGAGAACATC	61
*Ctsk*	GGGAGAAAAACCTGAAGC	ATTCTGGGGACTCAGAGC	61
*Acp5*	TGTGGCCATCTTTATGCT	GTCATTTCTTTGGGGCTT	61

### 2.9 Dual‐Luciferase Reporter Assay

RAW264.7 cells stably expressing NFATc1 or NF‐κB luciferase reporters were pre‐incubated with SOL for 1 h before exposure to RANKL (100 ng/mL). Luciferase activity was measured after 6 h (for NF‐κB) or 24 h (for NFATc1) using the Luciferase Assay System (Promega, Sydney, NSW, Australia).

### 2.10 Western Blot

Protein lysates were prepared using RIPA buffer. Equal amounts of protein were fractionated by SDS‐PAGE and transferred to PVDF membranes (Cytiva, Silverwater, NSW, Australia). Following antibody incubation, protein bands were visualized using ECL reagents (Amersham Pharmacia Biotech) and captured with the ImageQuant LAS 4000 system (Cytiva).

### 2.11 Intracellular ROS Measurement

Intracellular ROS production was detected using the DCFH‐DA probe (Abcam, Melbourne, VIC, Australia). BMMs were exposed to RANKL and SOL for 72 h, then loaded with 10 μM DCFH‐DA in darkness for 30 min. Fluorescence images were acquired via confocal microscopy.

### 2.12 Live‐Cell Calcium Imaging

BMMs were seeded in 48‐well plates and treated for 24 h. Cells were then loaded with Fluo‐4 AM (Thermo Fisher Scientific) and incubated at 37°C for 45 min. Calcium flux was monitored using an inverted fluorescence microscope (Nikon, Tokyo, Japan). Images were recorded at 2 s intervals over a 2 min duration, and data were analysed using Nikon NIS‐Elements software.

### 2.13 Statistical Analysis

All quantitative data are presented as the mean ± standard deviation (SD) from at least three independent biological replicates. Statistical evaluations were performed using GraphPad Prism software (version 9.0; San Diego, CA, USA). For comparisons between two groups, an unpaired Student's *t*‐test was employed. For experiments involving multiple groups, a one‐way analysis of variance (ANOVA) followed by Tukey's post hoc test was utilized to determine significant differences. Numerical data from the bone resorption and calcium oscillation assays were analysed using ImageJ (NIH) and Nikon NIS‐Elements software, respectively. A *p*‐value of < 0.05 was considered statistically significant (*p < 0.05, **p < 0.01, ***p < 0.001).

## 3 Results

### 3.1 Prediction of Potential SOL Targets and PPI Network Construction

**FIGURE 1:**
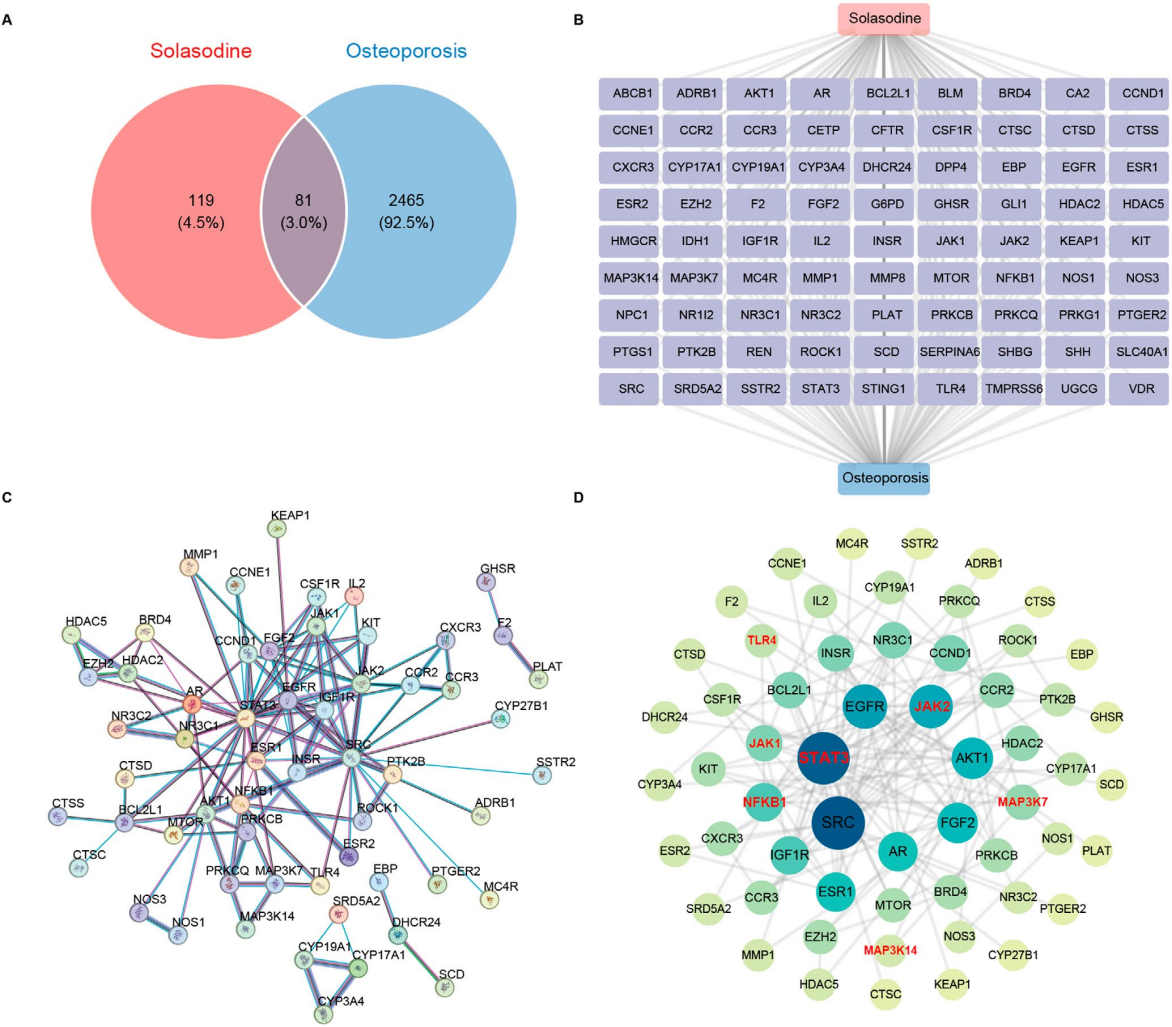
Network pharmacology analysis of SOL in osteoporosis. (A, B) 81 intersection targets between SOL and osteoporosis‐related genes. (C, D) Protein–protein interaction (PPI) network of the shared targets between SOL and osteoporosis; node size and colour gradient represent the degree value of each target.

To systematically identify the pharmacological targets of SOL, we integrated data from the SEA, SUPERPRED, HERB, and SwissTargetPrediction databases, yielding 200 potential candidate targets. Concurrently, 2546 osteoporosis‐related genes were retrieved from GeneCards, OMIM, and DisGeNET. Venn analysis revealed 81 overlapping targets between SOL and osteoporosis (Figure 1A,B), suggesting that these targets may serve as key nodes in SOL‐mediated therapeutic effects. To elucidate the functional interplay among these targets, an 81‐node Protein–Protein Interaction (PPI) network was constructed via the STRING database and visualized using Cytoscape. The resulting network comprised 81 nodes and 565 edges (Figure 1C,D), with topological analysis indicating a highly interconnected architecture, signifying that SOL likely exerts its anti‐osteoclastogenic effects through a multi‐target synergistic mechanism.

### 3.2 GO and KEGG Enrichment Analyses

**FIGURE 2:**
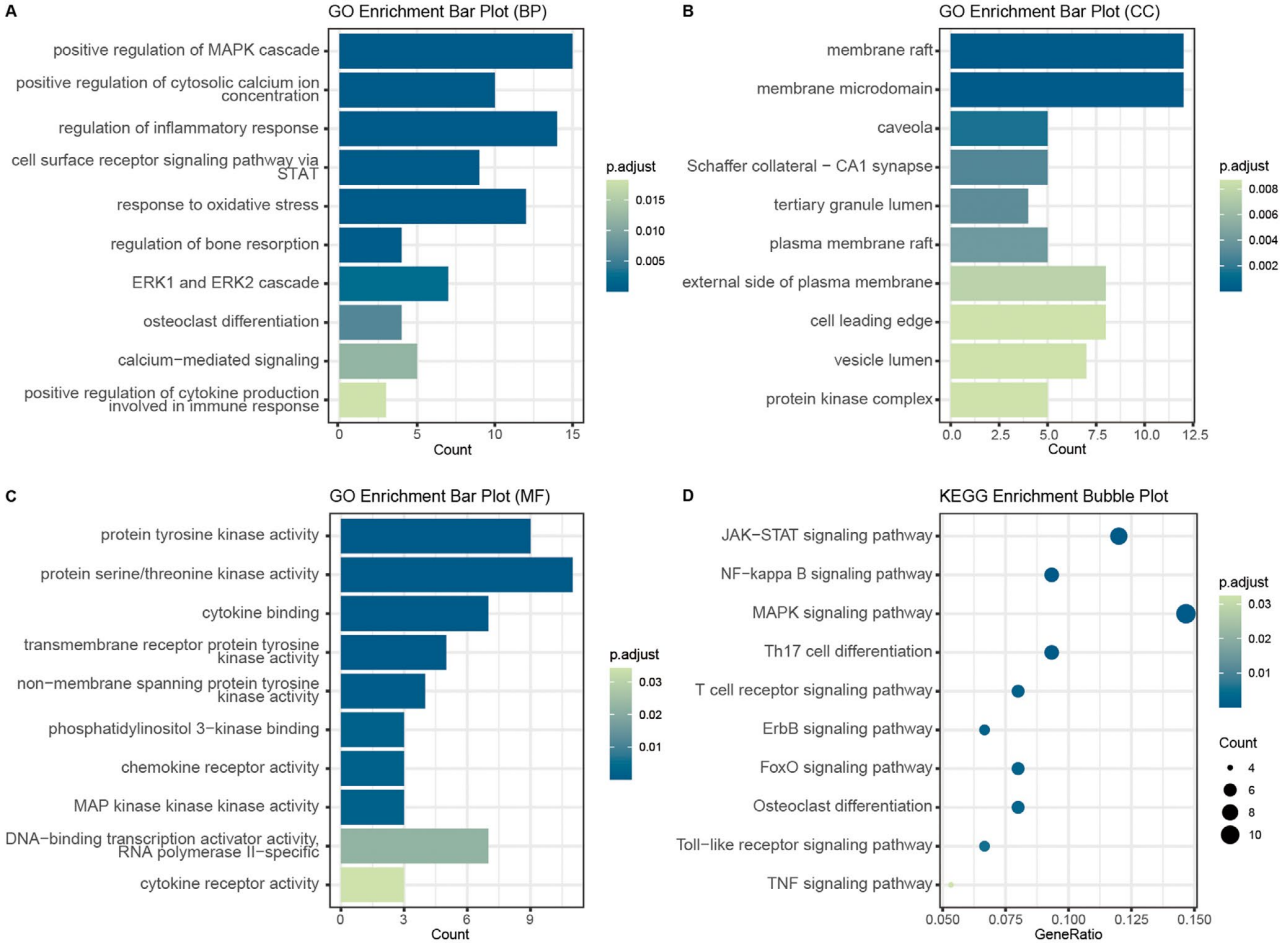
Functional enrichment analysis of intersection targets. (A) GO enrichment analysis of overlapping targets in Biological Processes (BP) (*p* < 0.05). (B) GO enrichment analysis of overlapping targets in Cellular Components (CC) (*p* < 0.05). (C) GO enrichment analysis of overlapping targets in Molecular Functions (MF) (*p* < 0.05). (D) Bubble plot representing the KEGG pathway enrichment analysis (*p* < 0.05).

To decode the molecular mechanisms underlying SOL's action, Gene Ontology (GO) and KEGG pathway analyses were performed on the 81 intersection targets. GO enrichment results (Figure 2A–C) revealed that in the Biological Process (BP) category, targets were predominantly involved in the regulation of the MAPK cascade, inflammatory response, oxidative stress, and calcium ion concentration. Cellular Component (CC) analysis highlighted enrichment in membrane rafts and protein kinase complexes, while Molecular Function (MF) terms were centered on protein serine/threonine kinase activity and cytokine receptor binding. KEGG analysis (Figure 2D) demonstrated significant enrichment in pathways pivotal to osteoclastogenesis, including the MAPK, NF‐κB, JAK–STAT, TNF, and Toll‐like receptor signalling pathways. These pathways serve as critical downstream effectors of the RANKL signalling axis.

### 3.3 Validation of Compound‐Target Interactions via Molecular Docking

**FIGURE 3:**
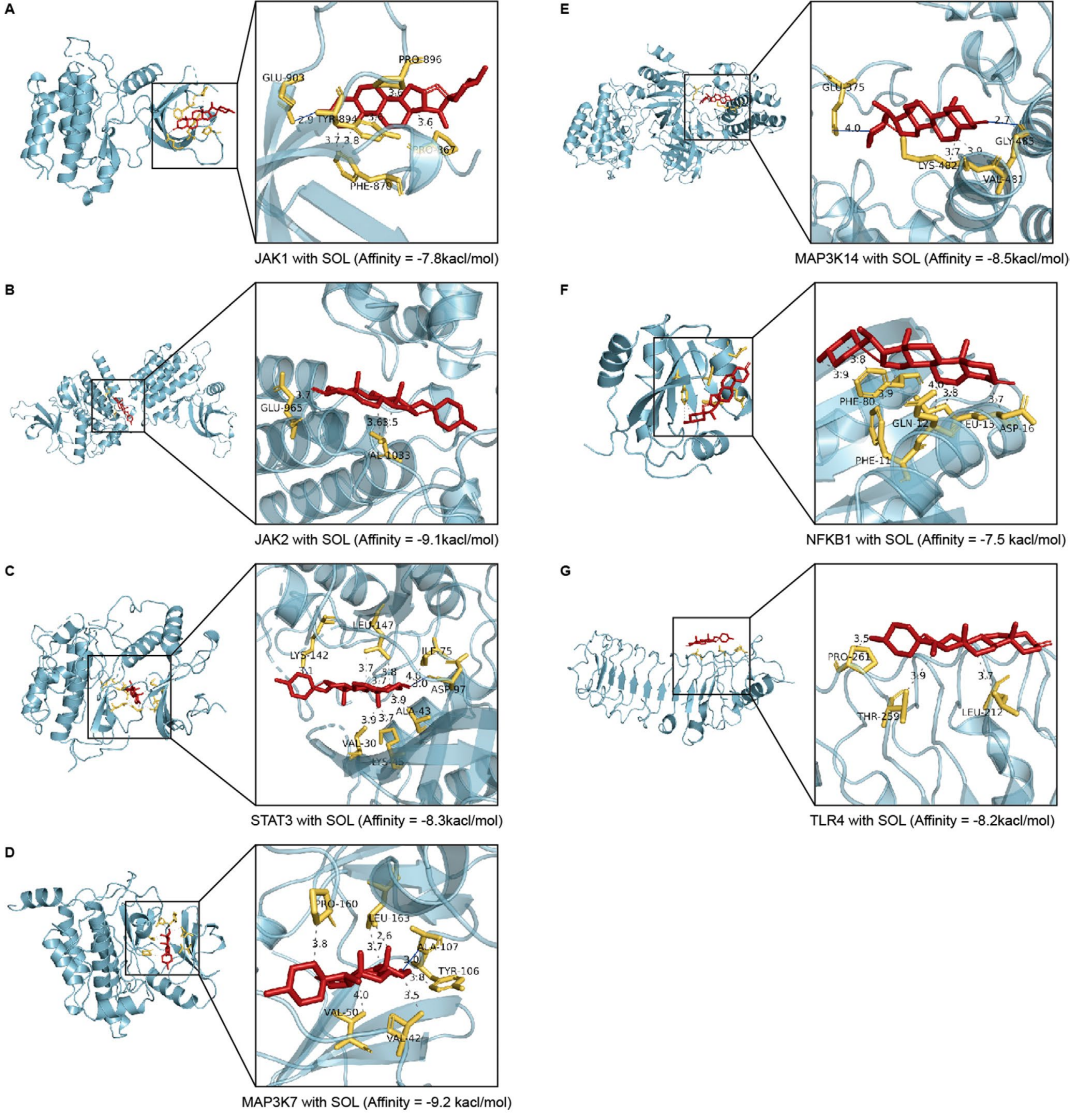
Molecular docking validation. Molecular docking simulations and binding energy analysis of SOL with key target proteins, including JAK1, JAK2, STAT3, MAP3K7, MAP3K14, NFKB1, and TLR4.

Guided by the bioinformatic findings, NFκB1, JAK1, JAK2, MAP3K7, MAP3K14, STAT3, and TLR4 were selected as core hubs for molecular docking validation. A binding energy (ΔG) ≤ −7.0 kcal/mol is typically indicative of a strong and stable ligand‐receptor interaction. Our simulations demonstrated that SOL exhibited robust binding affinities for all key target proteins, with binding energies consistently below −7.0 kcal/mol (Figure 3). These results corroborate the high‐confidence interactions predicted by network pharmacology.

### 3.4 SOL Attenuates RANKL‐Induced Osteoclastogenesis Without Cytotoxicity

**FIGURE 4:**
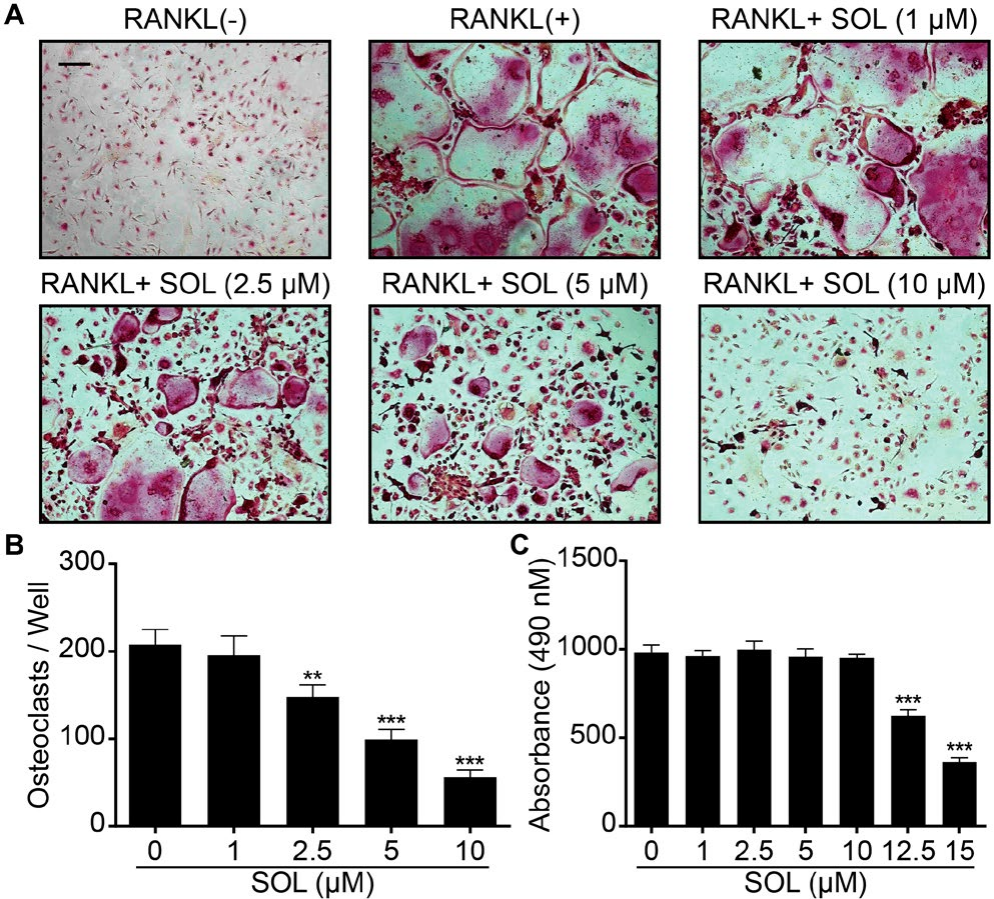
SOL suppresses RANKL‐induced osteoclastogenesis. (A) Representative images of TRAcP‐stained bone marrow‐derived macrophages (BMMs) following induction with different concentrations of SOL for 5 days. Scale bar = 200 μm. (B) Quantitative analysis of TRAcP‐positive multinucleated osteoclasts (nuclei ≥ 3). (C) Assessment of SOL cytotoxicity on BMMs via the MTS assay. Results are expressed as the mean ± SD of three independent experiments. Significant differences between groups are indicated as **p < 0.01 or ***p < 0.001.

**FIGURE 5:**
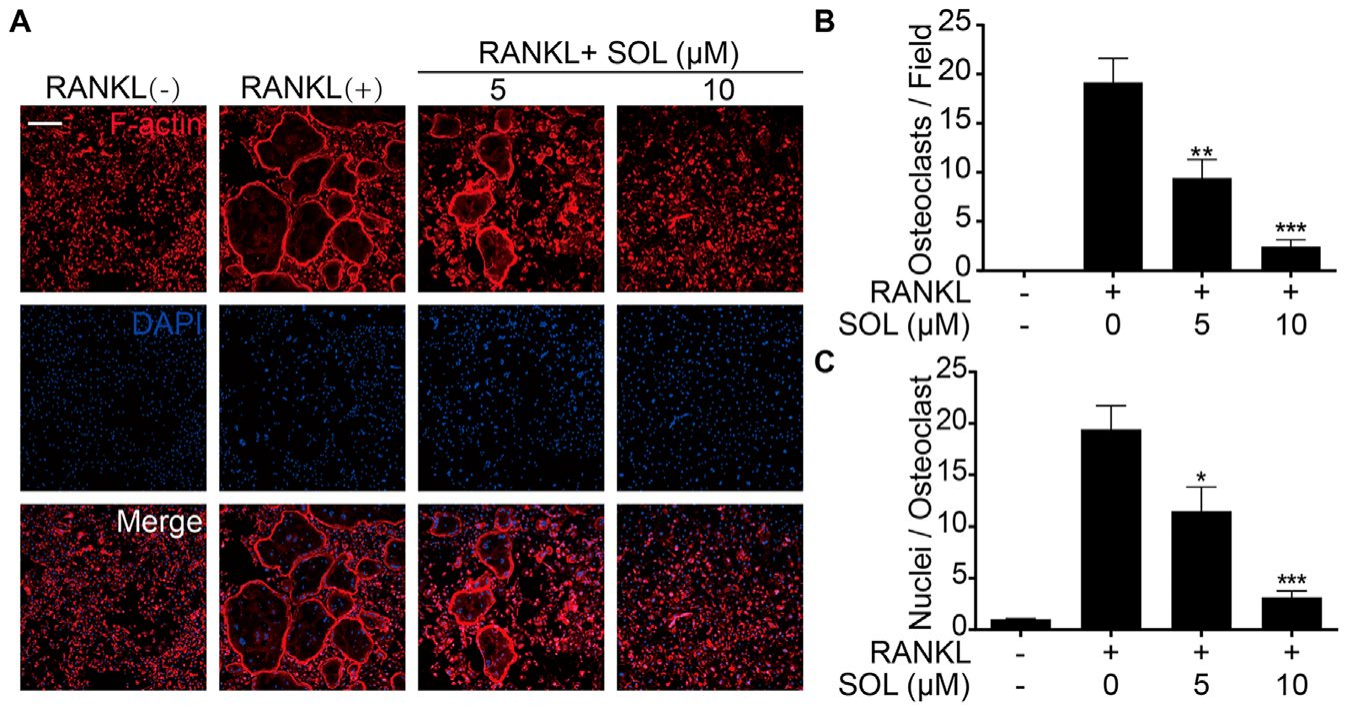
Sol inhibits RANKL‐induced osteoclast fusion. (A) Representative confocal images of osteoclast F‐Actin rings (rhodamine‐phalloidin, red) and nuclei (DAPI, blue). Scale bar = 200 μm. (B) Quantification of the number of osteoclasts per field. (C) Quantification of the average number of nuclei per osteoclast. Results are expressed as the mean ± SD of three independent experiments. Significant differences between groups are indicated as *p < 0.05, **p < 0.01, or ***p < 0.001.

To evaluate the impact of SOL on osteoclast differentiation, BMMs were stimulated with M‐CSF and RANKL in the presence of various SOL concentrations. TRAcP staining demonstrated that SOL dose‐dependently inhibited osteoclast formation; at concentrations ≥ 2.5 μM, both the number and size of multinucleated TRAcP‐positive cells were significantly reduced (Figure 4A,B). MTS assays confirmed that SOL exhibited no significant cytotoxicity toward BMMs at concentrations up to 10 μM (Figure 4C), suggesting its inhibitory effect is not due to compromised cell viability. Furthermore, Phalloidin and DAPI staining revealed that 5 μM and 10 μM SOL treatments severely disrupted F‐actin ring integrity and reduced the number of nuclei per cell (Figure 5), indicating an impairment of osteoclast fusion.

### 3.5 SOL Impairs Osteoclast Resorption Activity

**FIGURE 6:**
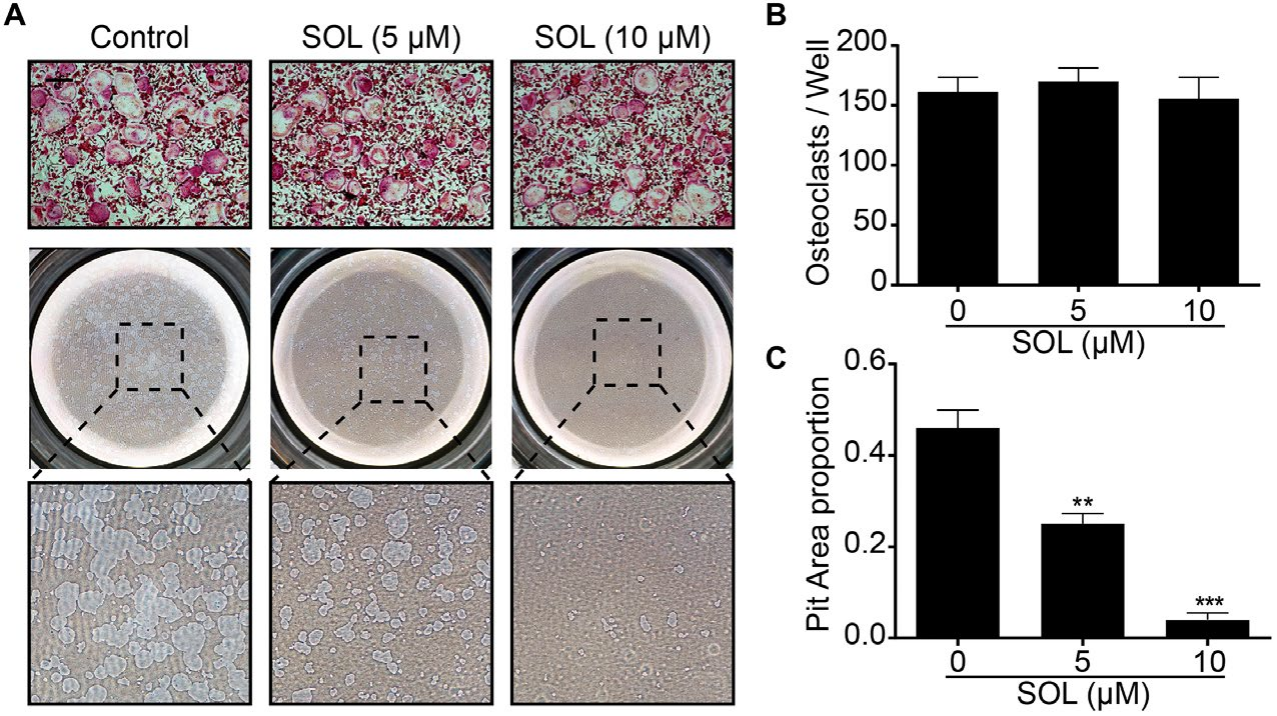
SOL impairs the bone resorptive function of osteoclasts. (A) Representative images of resorption pits on hydroxyapatite surfaces (middle and lower panels) and corresponding TRAcP‐stained osteoclasts (upper panels). Scale bar = 200 μm. (B) Quantification of the number of osteoclasts per well. (C) Quantification of the percentage of resorbed area on the hydroxyapatite surface. Results are expressed as the mean ± SD of three independent experiments. Significant differences between groups are indicated as **p < 0.01 or ***p < 0.001.

We further assessed the functional capacity of mature osteoclasts using a hydroxyapatite resorption assay. While SOL treatment did not significantly alter the survival count of pre‐differentiated mature osteoclasts (Figure 6B), it dramatically diminished the total resorbed area on the hydroxyapatite matrix in a dose‐dependent manner (Figure 6A,C). These data indicate that SOL effectively suppresses the bone‐resorbing activity of mature osteoclasts.

### 3.6 SOL Downregulates Osteoclast‐Specific Gene and Protein Expression

**FIGURE 7:**
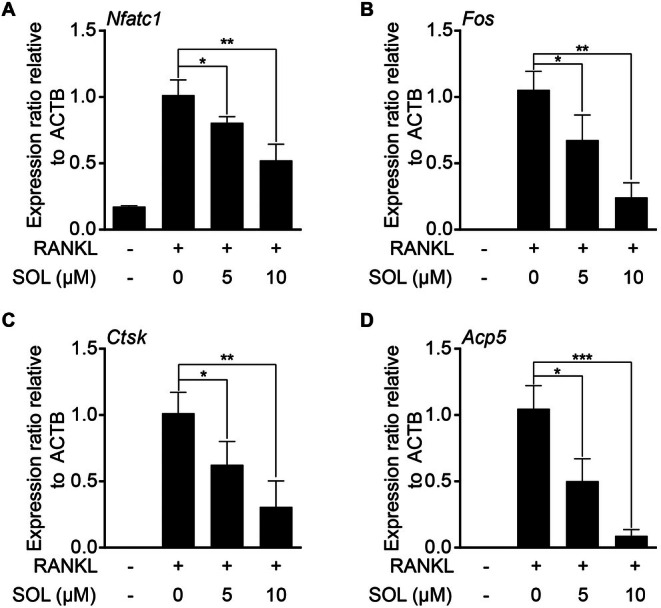
SOL downregulates RANKL‐induced osteoclastogenic gene expression. (A–D) Relative mRNA expression levels of Nfatc1, Fos, Ctsk, and Acp5 (TRAcP) in BMMs as detected by RT‐qPCR. Transcript levels were normalized to ACTB. Results are expressed as the mean ± SD of three independent experiments. Significant differences between groups are indicated as *p < 0.05, **p < 0.01, or ***p < 0.001.

**FIGURE 8:**
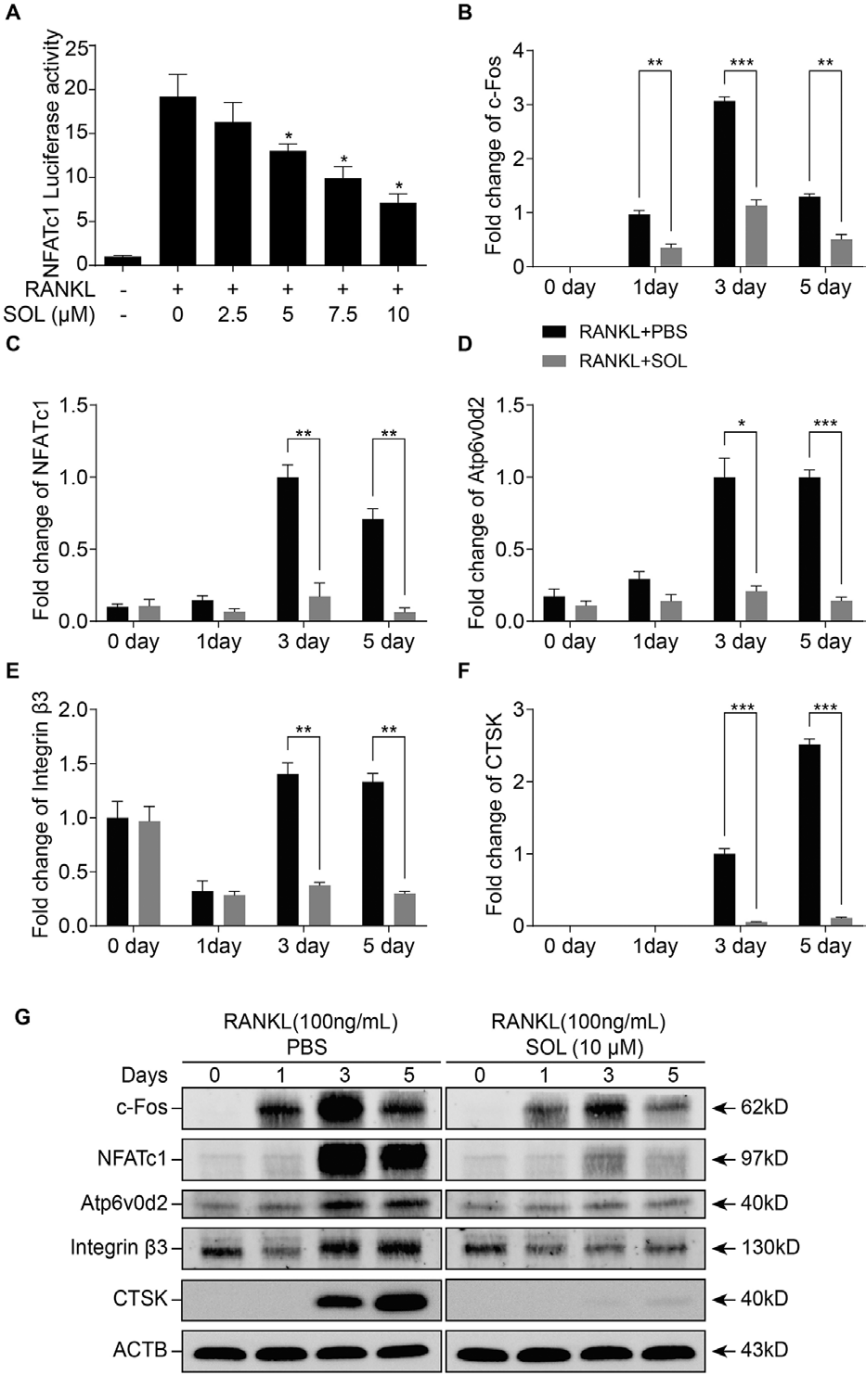
SOL inhibits NFATc1 activation and downstream protein expression. (A) Luciferase activity in RAW264.7 cells transfected with an NFATc1 reporter construct following RANKL stimulation and SOL pretreatment. (B–G) Representative Western blot images and quantitative analysis of c‐Fos, NFATc1, Atp6v0d2, Integrin β3, and CTSK protein levels. ACTB served as the loading control. Results are expressed as the mean ± SD of three independent experiments. Significant differences between groups are indicated as *p < 0.05, **p < 0.01, or ***p < 0.001.

RT‐qPCR analysis revealed that SOL (5 and 10 μM) significantly suppressed the RANKL‐induced mRNA expression of key osteoclastogenic markers, including Nfatc1, Fos (c‐Fos), Ctsk, and Acp5 (TRAcP) (Figure 7). The primer sequences used for RT‐qPCR are listed in Table 1. Luciferase reporter assays further demonstrated that SOL (≥ 5 μM) markedly inhibited the transcriptional activity of NFATc1 (Figure 8A). These findings were corroborated at the protein level via Western blotting, where 10 μM SOL treatment significantly reduced the expression of NFATc1 and c‐Fos, as well as their downstream targets Atp6v0d2, Integrin β3, and CTSK (Figure 8B–G).

### 3.7 SOL Inhibits the MAPK, STAT3, and NF‐κB Signalling Pathways

**FIGURE 9:**
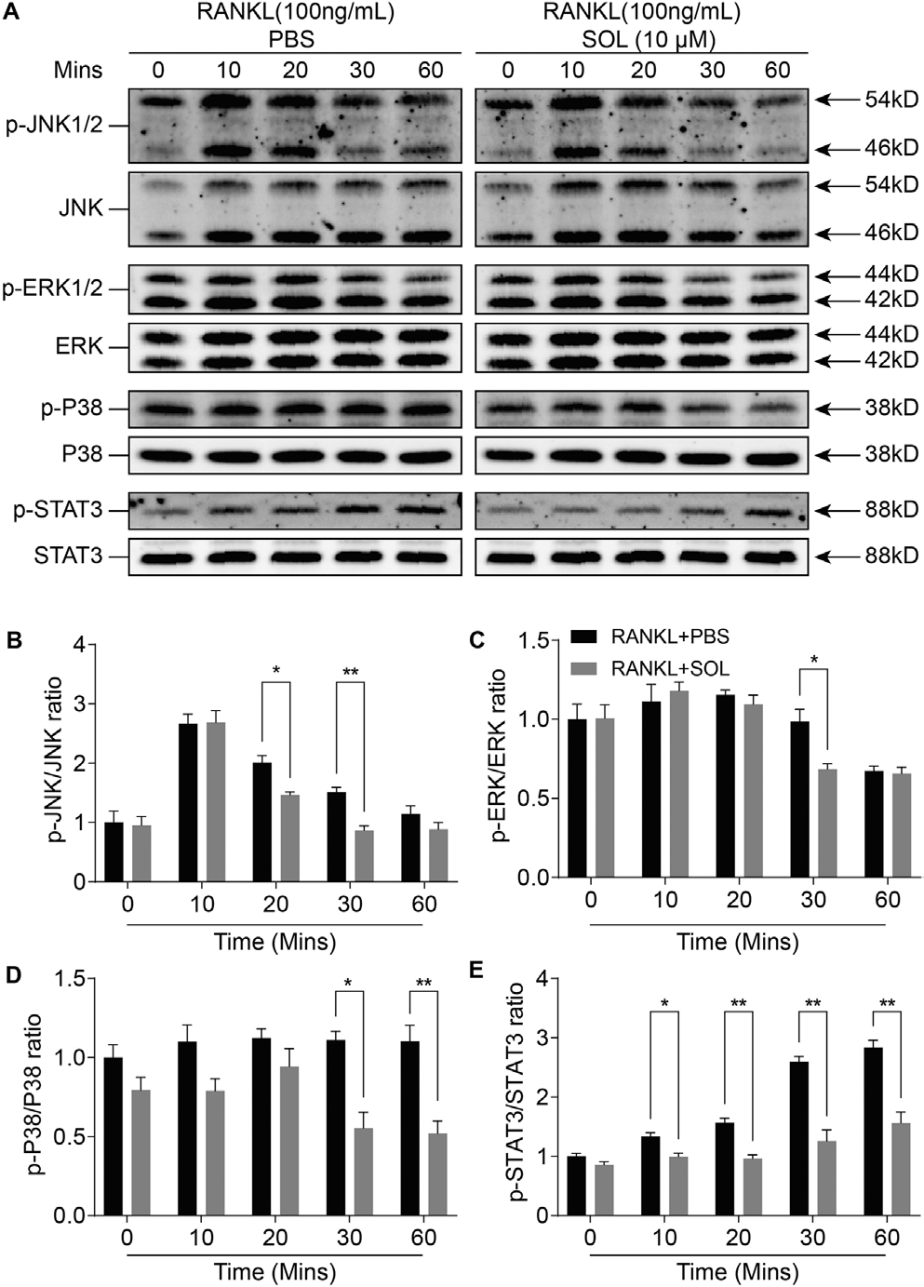
SOL attenuates the activation of MAPK and STAT3 signalling pathways. (A) Western blot images showing the effects of SOL on RANKL‐induced phosphorylation of p‐P38, P38, p‐JNK, JNK, p‐ERK, ERK, p‐STAT3, and STAT3. (B‐E) Quantitative analysis of phosphorylated protein levels relative to their respective total protein levels. Results are expressed as the mean ± SD of three independent experiments. Significant differences between groups are indicated as *p < 0.05 or **p < 0.01.

**FIGURE 10:**
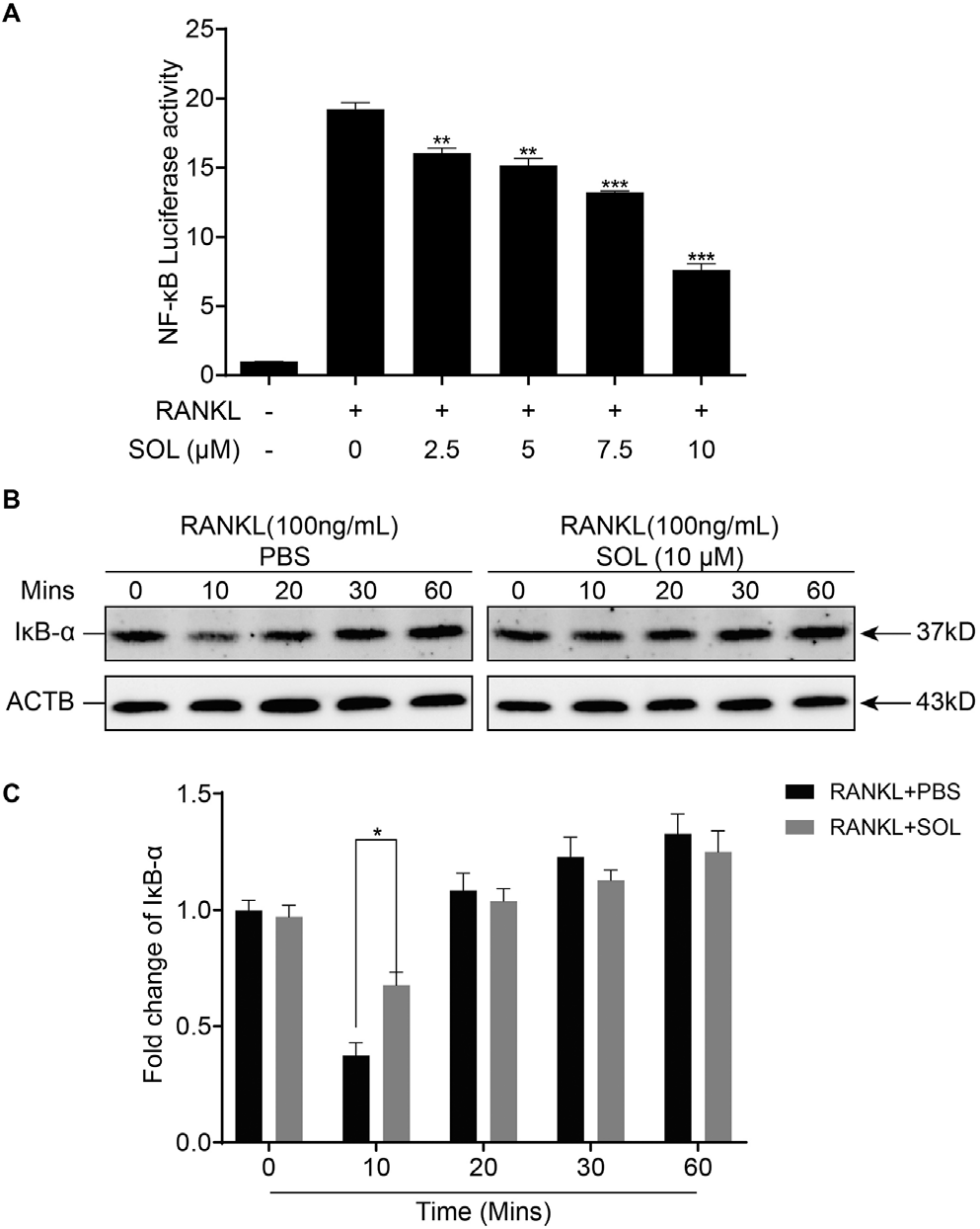
SOL disrupts RANKL‐induced NF‐κB pathway activation. (A) Luciferase activity in RAW264.7 cells transfected with an NF‐κB reporter construct. (B‐C) Representative Western blot images and quantitative analysis demonstrating the inhibitory effect of SOL on RANKL‐induced IκB‐α degradation. Results are expressed as the mean ± SD of three independent experiments. Significant differences between groups are indicated as *p < 0.05, **p < 0.01, or ***p < 0.001.

In alignment with our network pharmacology predictions, we investigated the effect of SOL on early signalling events. Western blot analysis showed that RANKL‐induced phosphorylation of p38, JNK, and ERK was significantly attenuated by SOL treatment (Figure 9A–E). Additionally, SOL (≥ 2.5 μM) significantly lowered NF‐κB luciferase reporter activity (Figure 10A) and effectively prevented the RANKL‐induced degradation of IκB‐α (Figure 10B,C). Furthermore, the activation of the STAT3 pathway was notably suppressed. These results suggest that SOL acts early in the signalling cascade to disrupt the major pathways required for NFATc1 induction.

### 3.8 SOL Reduces RANKL‐Induced ROS Generation

**FIGURE 11:**
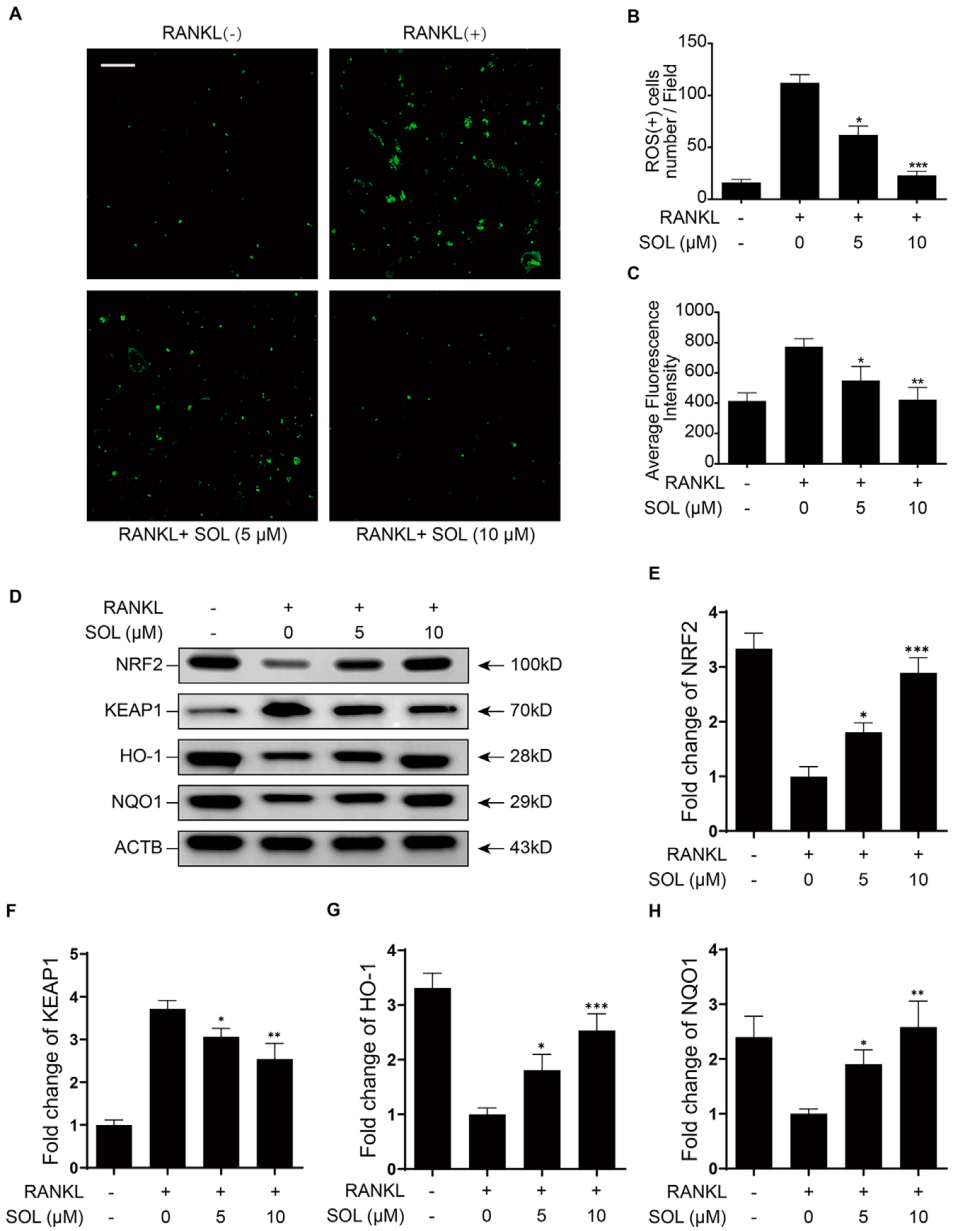
SOL mitigates RANKL‐induced ROS generation and regulates the Nrf2/Keap1 antioxidant signalling pathway during osteoclastogenesis. (A) Representative fluorescence images of ROS production in BMMs using the DCFH‐DA probe. Scale bar = 200 μm. (B) Quantification of the number of ROS‐positive cells per field. (C) Quantification of the mean fluorescence intensity (MFI) per cell. (D) Western blot analysis of NRF2, KEAP1, HO‐1, and NQO1 in BMMs under RANKL stimulation with or without SOL treatment. (E–H) Quantification of protein levels shown in (D). Results are expressed as the mean ± SD of three independent experiments. Significant differences between groups are indicated as *p < 0.05, **p < 0.01, or ***p < 0.001.

Since ROS serve as pivotal second messengers in osteoclast survival and differentiation, we utilized the DCFH‐DA probe to detect intracellular ROS levels. RANKL stimulation led to a marked surge in ROS production, which was effectively curtailed by 5 and 10 μM SOL treatment (Figure 11A). Both the percentage of ROS‐positive cells and the mean fluorescence intensity exhibited a dose‐dependent decline (Figure 11B,C), indicating that SOL exerts its anti‐osteoclastogenic effects partly by mitigating oxidative stress. ROS scavenging primarily depends on various antioxidant enzymes regulated by the Nrf2/Keap1 signalling axis. To further investigate how SOL reduces ROS levels, we examined the protein expression of Keap1 and Nrf2. Our results showed that under RANKL stimulation, Nrf2 expression in BMMs decreased, whereas the expression of its cytosolic inhibitor, Keap1, was increased. Notably, SOL treatment led to an increase in Nrf2 expression alongside a further reduction in Keap1 levels (Figure 11D–F). Finally, we evaluated the expression of key antioxidant enzymes, such as heme oxygenase‐1 (HO‐1) and NADPH quinone oxidoreductase 1 (NQO1). As shown in Figure 11D,G,H, protein levels of HO‐1 and NQO1 were significantly downregulated following RANKL stimulation compared to the control group, an effect that was effectively reversed by SOL treatment. These findings suggest that SOL attenuates oxidative stress, which is associated with the modulation of the Nrf2/Keap1 antioxidant defence system.

### 3.9 SOL Abrogates RANKL‐Induced Ca^2+^ Oscillations and CaMKIV Activation

**FIGURE 12:**
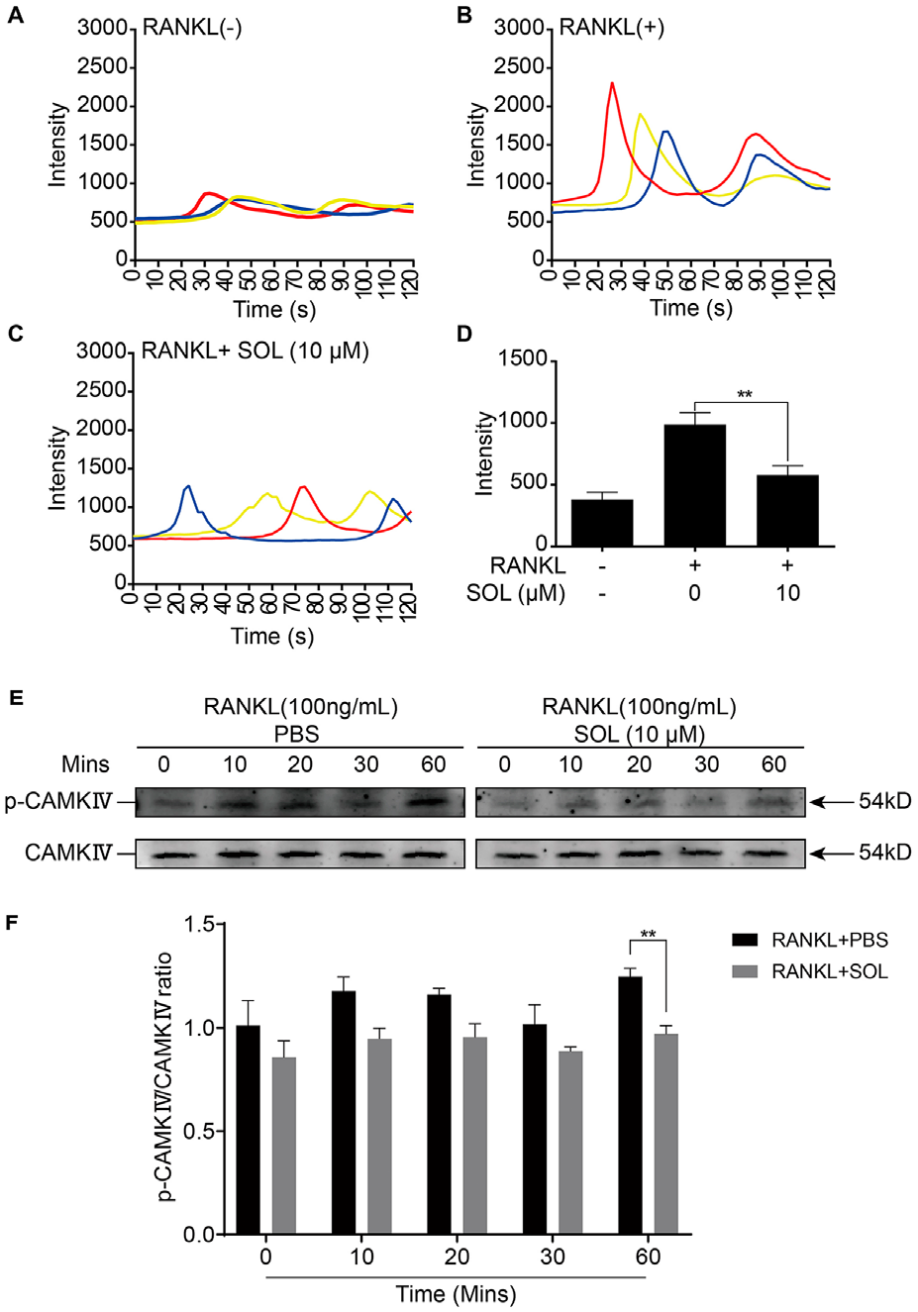
SOL inhibits RANKL‐induced Ca^2+^ oscillations and CaMKIV activation. (A–C) Representative intracellular Ca^2+^ oscillation traces in BMMs from the Control, RANKL‐induced, and SOL‐treated groups. (D) Quantitative analysis of the amplitude/intensity of single‐cell Ca^2+^ oscillations. (E, F) Representative Western blot images and quantitative analysis of CaMKIV phosphorylation (p‐CaMKIV) relative to total CaMKIV. Results are expressed as the mean ± SD of three independent experiments. Significant differences between groups are indicated as **p < 0.01.

To explore the influence of SOL on calcium signalling, we monitored live‐cell Ca^2+^ flux. RANKL stimulation triggered vigorous intracellular Ca^2+^ oscillations, whereas SOL treatment significantly reduced the amplitude and frequency of these oscillations (Figure 12A–D). Consistently, Western blotting demonstrated that SOL significantly inhibited the phosphorylation of CaMKIV, a critical downstream effector of the calcium signalling pathway (Figure 12E,F).

## 4 Discussion

The aberrant activation of osteoclast‐mediated bone resorption is a critical factor in the pathogenesis of metabolic bone diseases such as osteoporosis [1]. SOL, a natural steroidal alkaloid derived from the Solanaceae family, is widely utilized in the pharmaceutical industry due to its structural homology with endogenous steroidal hormones [20]. While the anti‐inflammatory and antioxidant properties of SOL have been documented [21], its specific regulatory role in bone metabolism has not been systematically investigated prior to this study. In this study, we systematically evaluated the anti‐osteoclastogenic potential of SOL. Our findings demonstrate that SOL effectively suppresses RANKL‐induced osteoclast differentiation and resorptive function by coordinately modulating multiple intracellular signalling cascades, including the MAPK, NF‐κB, and STAT3 pathways, while simultaneously attenuating ROS production and Ca^2+^ oscillations.

Network pharmacology provides a systematic framework for deciphering the “drug–target–pathway” interactome of natural compounds. Our bioinformatic analysis identified 81 common targets between SOL and OP, with functional enrichment highlighting critical cascades such as the MAPK, NF‐κB, and JAK–STAT signalling pathways. Molecular docking further substantiated these findings, revealing high‐confidence binding affinities between SOL and hub proteins like JAK1, STAT3, and MAP3K7. These in silico predictions provided a robust theoretical roadmap for our subsequent experiments, suggesting that SOL likely acts through a polypharmacological mechanism, exerting synergistic orchestration over multiple signalling hubs rather than a single‐molecule interaction.

Osteoclastogenesis involves multiple stages, including progenitor cell proliferation, fusion, and functional maturation. The fusion of mononuclear precursors into giant multinucleated cells under RANKL induction is a hallmark of functional bone resorption. This process is regulated by fusion‐related molecules such as DC‐STAMP, OC‐STAMP, and Atp6v0d2 [22, 23, 24]. Notably, Atp6v0d2 plays a vital role in mediating cell membrane fusion, and its deficiency leads to impaired osteoclast fusion [24]. At the functional level, mature osteoclasts degrade the bone matrix by secreting enzymes such as CTSK and Acp5 [25]. This process depends on the adhesion of osteoclasts to the bone matrix mediated by Integrin β3, which facilitates the formation of the sealing zone—a specialized osteoclast‐matrix interface essential for localized microenvironmental acidification [26]. Furthermore, the hydroxyapatite resorption assay revealed a marked, dose‐dependent decrease in the total resorbed area following SOL intervention, which was corroborated by the downregulation of key markers including Atp6v0d2, Integrin β3, CTSK, and Acp5. Collectively, these findings suggest that SOL suppresses the differentiation and resorptive activity of osteoclasts by downregulating the expression of proteins essential for precursor fusion, matrix adhesion, and osteolytic function.

At the molecular level, NFATc1 and c‐Fos are pivotal transcription factors regulating the expression of osteoclast‐specific genes [15, 27]. Upon RANKL activation, c‐Fos participates in the formation of the AP‐1 complex to initiate the transcription priming of *Nfatc1*. Subsequently, NFATc1 maintains high expression levels through a positive feedback auto‐amplification loop and drives the transcription of downstream functional genes, including *Ctsk*, *Acp5*, and *Atp6v0d2* [15, 28, 29]. Our experiments confirmed that SOL downregulated both the mRNA and protein expression of NFATc1 and c‐Fos and inhibited the transcriptional activity of NFATc1. These results indicate that the anti‐osteoclastogenic effect of SOL is mediated, at least in part, by the direct blockade of the central c‐Fos/NFATc1 regulatory axis.

Further mechanistic investigations revealed that SOL exerts a broad regulatory effect on the complex signalling networks mediated by RANKL. In classical kinase pathways, the MAPK family (p38, ERK, and JNK) regulates AP‐1 activity: ERK phosphorylation directly modulates c‐Fos transcription, while JNK enhances AP‐1 activity via c‐Jun phosphorylation [30, 31, 32]. Simultaneously, the NF‐κB pathway facilitates nuclear translocation through the degradation of IκB‐α, an essential early step in the osteoclast differentiation program [33]. Moreover, STAT3, a core member of the JAK–STAT pathway, has recently been shown to act as a crucial regulator of pathological bone resorption [34]. In alignment with these bioinformatic predictions, our results showed that SOL significantly reduced the phosphorylation levels of p38, ERK, JNK, and STAT3, and effectively inhibited the degradation of IκB‐α. The simultaneous inhibition of these disparate yet interconnected pathways indicates that SOL serves as a comprehensive modulator of the early signalling events and kinase‐driven commitment required for the osteoclast lineage.

Beyond protein kinase cascades, the role of intracellular signalling mediators—specifically ROS and Ca^2+^ oscillations—is critical for sustained osteoclast maturation. This functional focus is consistent with our GO enrichment results, which emphasized “positive regulation of cytosolic calcium ion concentration”, “calcium‐mediated signalling” and “response to oxidative stress” as key biological processes regulated by SOL intersection targets. RANKL‐induced ROS generation, primarily mediated by NADPH oxidase, serves as a synergistic signal that intensifies the activation of MAPK and NF‐κB by inactivating dual‐specificity phosphatases that would otherwise terminate these kinase signals [35]. Under physiological conditions, Keap1 anchors Nrf2 in the cytoplasm for proteasomal degradation. Upon activation, Nrf2 translocates to the nucleus to trigger the expression of antioxidant enzymes such as HO‐1, GSR, NQO1, and CAT [36]. Recent studies have shown that various small molecules inhibit osteoclastogenesis by modulating the Nrf2/Keap1 axis to enhance antioxidant defences, such as 4‐methylcatechol and Notopterol [37, 38]. Our findings suggest that SOL reduces Keap1 levels and enhances Nrf2 protein accumulation, thereby upregulating the expression of antioxidant enzymes and reducing intracellular ROS levels. Simultaneously, characteristic Ca^2+^ oscillations promote NFATc1 dephosphorylation via calcineurin and induce CaMKIV to phosphorylate CREB, driving the sustained auto‐amplification of *Nfatc1* [39]. Our data indicated that SOL suppressed both the amplitude of Ca^2+^ oscillations and the phosphorylation of CaMKIV. This coordinated regulation indicates that SOL interferes with multiple signalling networks required for osteoclast development.

Osteoblasts, derived from bone marrow mesenchymal stem cells, play a fundamental role in bone formation. Bone homeostasis is determined by the dynamic balance between osteoclastic resorption and osteoblastic formation [40]. Therefore, evaluating SOL solely in osteoclast models provides an incomplete picture of its therapeutic potential. While the present study focused on the anti‐catabolic effects of SOL, it is noteworthy that numerous alkaloids, such as Berberine and Matrine, exert both anti‐resorptive and pro‐anabolic effects in maintaining bone homeostasis [41, 42, 43, 44, 45, 46]. Given that SOL shares structural similarity with endogenous steroidal hormones and exhibits antioxidant activity, it is plausible that it may enhance osteoblast proliferation and differentiation via modulation of oxidative stress. Future studies combining in vitro osteoblast assays with in vivo animal models are required to comprehensively elucidate the role of SOL in modulating the balance of bone metabolism.

## 5 Conclusion

**FIGURE 13:**
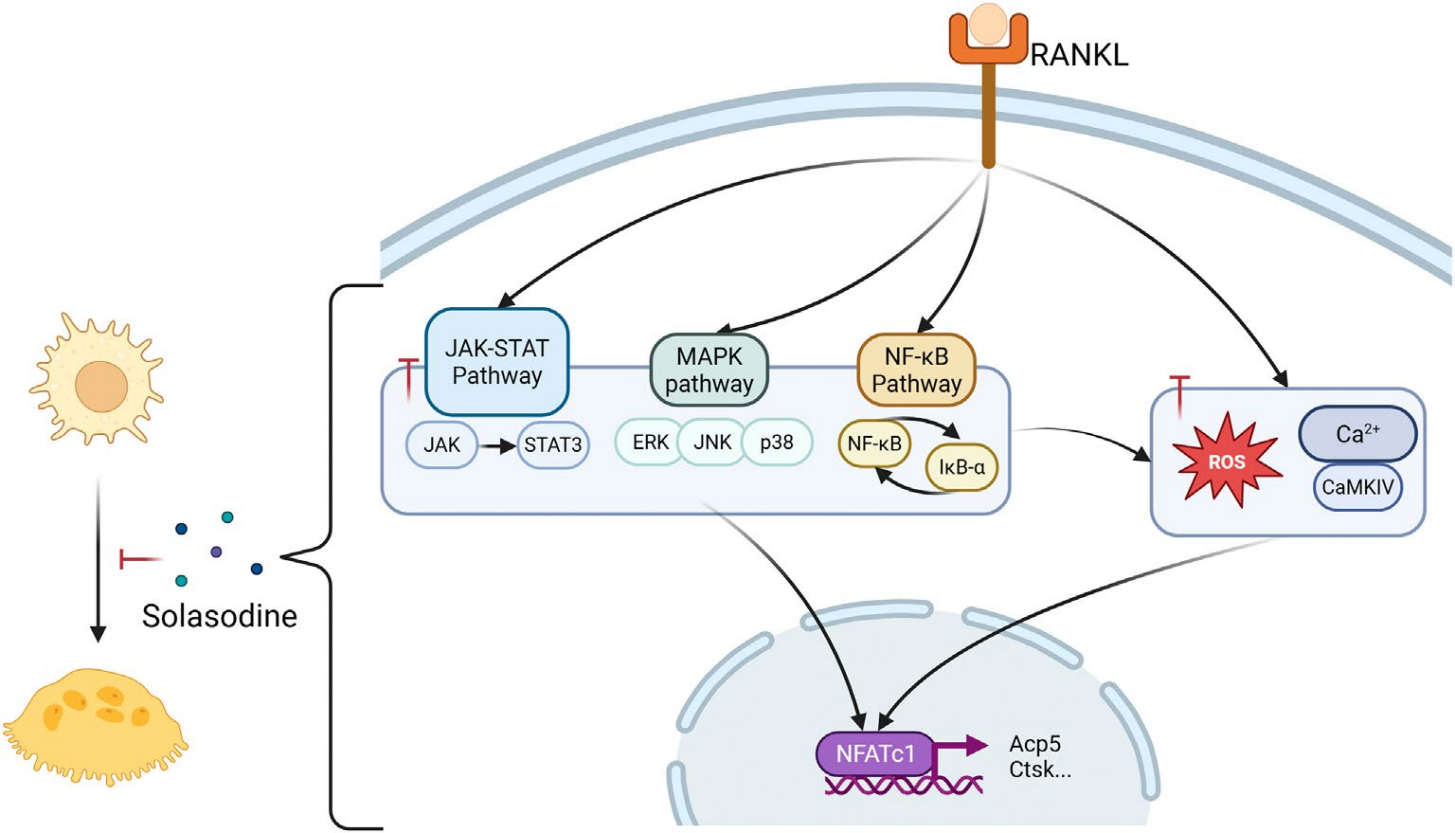
Schematic diagram of the role of SOL in osteoclast formation and function. SOL attenuates NFATc1 activation and expression by modulating the MAPK, STAT, and NF‐κB pathways, ROS levels, and intracellular Ca^2+^ oscillations, thereby inhibiting osteoclastogenesis and bone resorption. Figure created with BioRender.

In conclusion, our findings suggest that SOL inhibits RANKL‐induced osteoclast formation and bone resorption by modulating the MAPK, NF‐κB, and JAK–STAT signalling pathways, as well as suppressing ROS generation and Ca^2+^ oscillations, ultimately leading to the downregulation of the NFATc1/c‐Fos axis. This multi‐dimensional regulatory effect collectively mediates the inhibitory action of SOL on osteoclastogenesis (Figure 13). As a natural compound with a characteristic steroidal scaffold, SOL exhibits significant potential for the prevention and treatment of bone diseases associated with aberrant osteoclast activity.

## Author Contributions

**Yiwei Jiang:** writing – original draft, methodology, investigation. **Maihuan Wang:** software, formal analysis. **Xiaoyi Ji:** software, formal analysis. **Zhiyu Jin:** writing – original draft, methodology. **Zhen Cao:** funding acquisition, visualization, supervision, writing – review and editing.

## Funding

The authors have nothing to report.

## Conflicts of Interest

The authors declare no conflicts of interest.

## Data Availability

The data that support the findings of this study are available from the corresponding author upon reasonable request.
